# IMU-Based Wearable Insoles in Clinical Settings: Key Parameters Differentiating Clinical and Non-Clinical Populations

**DOI:** 10.3390/s26061802

**Published:** 2026-03-12

**Authors:** Sheng Lin, Kerrie Evans, Dean Hartley, Scott Morrison, Stuart McDonald, Martin Veidt, Gui Wang

**Affiliations:** 1 School of Mechanical and Mining Engineering, The University of Queensland, St Lucia, QLD 4072, Australia; sheng.lin@uq.edu.au (S.L.); s.mcdonald1@uq.edu.au (S.M.); m.veidt@uq.edu.au (M.V.); 2 Healthia Limited, Bowen Hills, QLD 4006, Australia; 3 Faculty of Medicine and Health, The University of Sydney, Camperdown, NSW 2050, Australia; 4 iOrthotics Pty Ltd., Windsor, QLD 4030, Australia; 5 Henan Academy of Innovations in Medical Science, Zhengzhou 451162, China; 6 Nantong Institute of Advanced Technology, Shanghai Jiaotong University, Nantong 226300, China

**Keywords:** wearable insole device, gait analysis, inertial measurement unit, foot biomechanics

## Abstract

Wearable systems based on inertial measurement units (IMUs) have attracted considerable interest in recent years in the field of gait analysis. However, most gait studies using such devices have been conducted in laboratory rather than clinical settings. This study evaluated a commercially available IMU-based insole system in two cohorts: a clinical group (59 ± 18, years) recruited from podiatry clinics and a non-clinical group (28 ± 7, years) recruited from a university with no reported complaints. Participants wore the IMU-based device and performed treadmill walking (clinical group) and overground walking (non-clinical group). Spatiotemporal parameters were compared between groups using statistical analyses included the Shapiro–Wilk test, Mann–Whitney test, and Welch’s *t*-tests for non-bilateral data, and a two-factor linear mixed-effects model estimated by restricted maximum likelihood (REML) for bilateral spatiotemporal parameters to evaluate group, foot-side, and interaction effects. Ten of the twenty-two spatiotemporal parameters showed significant group differences, with statistical significance observed in at least one foot for parameters measured bilaterally. The observed differences may reflect a combination of clinical characteristics, age-related effects, and walking environment influences. Findings are discussed in relation to potential biomechanical mechanisms, factors influencing results and the clinical utility of IMU systems. Future research should investigate specific foot conditions under standardized walking conditions with age-matched cohorts.

## 1. Introduction

Gait analysis refers to the systematic study and characterization of human walking patterns [1,2], involving the quantitative assessment of biomechanical parameters such as spatiotemporal parameters. Accurate assessment of gait patterns and gait characteristics in the podiatry clinical setting can help identify foot dysfunction, support the delivery of effective and personalized treatment, inform predictive outcome assessment, and facilitate the application of precision medicine.

Currently, gait assessments in clinical settings often rely on observational methods, such as direct visual inspection or video analysis. These approaches are advantageous as they are quick, simple, and low cost compared to instrumented analysis [1]. However, the validity, reliability, and repeatability of traditional observational assessment tools have been questioned [3]. Clinical gait observation remains subjective, dependent on clinicians’ expertise and experience [3,4]. Subjective gait assessment poses limitations such as lacking sufficient quantitative data to support objective decision-making, highlighting a critical need for integrating more accurate, data-driven assessment approaches into clinical practice.

Quantitative, instrumented-based gait analysis is the most often conducted in laboratory settings in research institutions. For example, motion capture systems which use multiple thermal cameras or high-speed cameras, computer processing software, calibration hardware, etc., to measure gait activities [5]. In addition, pressure systems embed numerous pressure sensors to measure the kinetic data, like plantar pressure distribution or ground reaction forces, under static or dynamic conditions [6]. Although these techniques are well-established and widely recognized as the gold standard in gait research, they are not often used in clinical settings due to factors such as complex instrumentation and protocols or high costs. Moreover, data management and analytical processes in gait laboratories may differ significantly from clinical settings, where clinical teams often vary considerably in training and expertise, thus presenting additional challenges for the effective translation and implementation of these technologies into routine clinical practice.

With recent advancements in wearable technologies, wearable sensors have emerged as potential tools that can be introduced into clinical settings to quantitatively measure and characterize gait patterns, thereby providing objective data to support precise treatment by podiatrists. Among wearable sensors, inertial measurement units (IMUs), which integrate multiple sensors including accelerometers and gyroscopes, are gaining attention. Specifically, gyroscopes within IMUs typically measure angular motion through angular velocities and enable the accurate detection of key gait events and subsequently facilitate the extraction of essential temporal gait information [7]. Accelerometers, on the other hand, are primarily utilized to derive additional parameters such as velocity and displacement. Through sensor fusion techniques and advanced algorithms, IMUs can be used to determine reliable and meaningful spatiotemporal gait parameters essential for characterizing and quantifying gait features. IMU sensors have already been effectively applied within laboratory settings for various gait studies as highlighted in a recent review paper [8]. For instance, IMUs placed at different regions of the foot have successfully captured foot movements, allowing researchers to accurately quantify spatiotemporal gait parameters such as stride length, stance, swing durations, and gait speed throughout the gait cycle [9,10]. Additionally, IMUs have demonstrated utility in laboratory studies for characterizing pathological gait disorders or abnormalities associated with condition such as diabetes [11] and Parkinson’s disease [12].

Wearable sensors embedded into insoles have become a mainstream technology in the development of wearable devices for gait analysis [13,14]. Such smart insoles offer significant potential to enable remote health monitoring by capturing gait-related health metrics. Commercial IMU-based smart insoles, such as FeetMe^®^ [15,16], PODOSmart^®^ [17,18], and Digitsole Pro (DSPro^®^) [19], have emerged on the market. These commercial products have undergone validation studies aimed at evaluating their accuracy and reliability against gold standard laboratory-based gait analysis systems.

Quantitative spatiotemporal parameters derived from IMUs or IMU-based wearable devices have been applied in gait studies in controlled laboratory settings; however, there are limited reported instances of their application in clinical environments [20]. Although previous studies have demonstrated the capability of IMU sensors to distinguish between normal and abnormal gait patterns [21,22], these laboratory studies often focus on gait abnormalities associated with specific conditions. In contrast, within clinical settings, gait abnormality may be influenced by multiple factors not confined to specific pathologies, such as joint dysfunction, soft tissue changes, and pain on the foot plantar. Additionally, clinical gait measurement tools and indices commonly emphasize temporally related gait parameters [20], whereas angle parameters, particular those concerning frontal and transverse foot rotations, are rarely quantified, primarily due to the difficulty of capturing spatial foot movements in a clinical setting. Under such circumstances, it remains unclear which parameters from IMU-based device offer the ability to differentiate populations presenting for clinical examination with those in the broader community. Identifying which spatiotemporal parameters hold clinical value in distinguishing potential pathological gait, especially before examination by podiatrist, could therefore be highly beneficial, enabling podiatrists to prioritize these parameters for rapid assessment. Therefore, this study employed commercial IMU devices, DSPro^®^, in both clinical and non-clinical settings, to identify which spatiotemporal gait parameters can significantly differentiate the gait of a group presenting for clinical examination with one recruited from a population with no podiatry related complaints.

## 2. Methodology

### 2.1. Participants Recruitment

Participants were recruited into two groups. The control group consisted of individuals from a local university, while the clinical group was recruited from a local podiatry clinic, potentially presenting with a variety of complaints related to foot pain or disorders. Eligibility criteria for both groups included: (i) the ability to walk without the aid of any orthopedic device, and (ii) the feasibility of fitting the insole with IMUs into participants’ own footwear. For the clinical testing, eight podiatrists from the podiatry clinic conducted the assessments, whereas the university testing was carried out with the assistance of the authors. The study received approval from the Human Research Ethics Committee at the University of Queensland (reference number 2022/HE000445). Written informed consent was obtained from all participants prior to data collection. A summary of the recorded clinical characteristics, including reported pain location, joint range of motion, and presenting foot conditions, is provided in the Supplementary Materials.

### 2.2. Instruments

**Figure 1:**
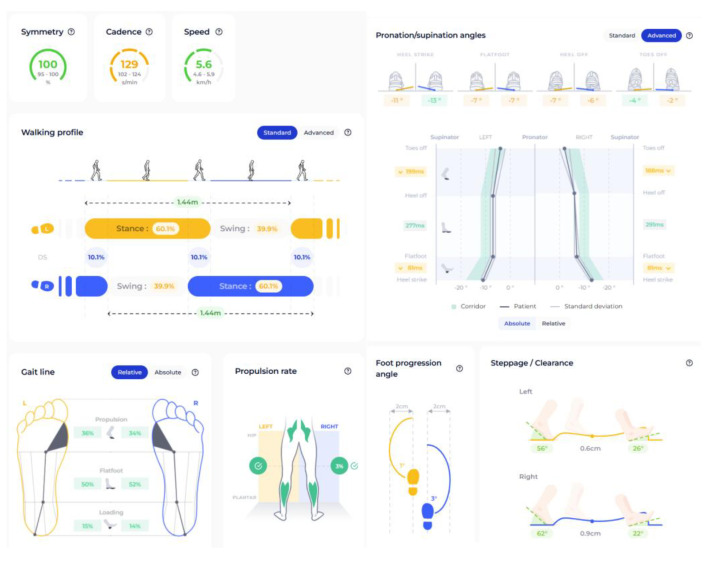
Graphical illustration of spatiotemporal parameters from DSPro^®^.

DSPro^®^ is a smart insole system for gait monitoring comprising insoles with detachable IMU sensors. The device connects via Bluetooth to a mobile app or web platform, where data can be uploaded to the cloud and the results are displayed. The DSPro^®^ system employs proprietary AI algorithms embedded within its Mov-Scan technology platform to derive spatiotemporal and kinematic parameters from raw inertial signals. Although the detailed algorithms are not publicly available, the reliability and validity of DSPro^®^ have been previously evaluated against laboratory-based motion capture systems [19]. A validation study reported good to excellent test–retest reliability for most spatiotemporal parameters, with intraclass correlation coefficients (ICCs) exceeding 0.75 during overground walking and 0.86 during treadmill walking across different speeds. Concurrent validity analyses demonstrated moderate to excellent agreement with motion capture references, with ICC values ranging from 0.673 to 0.999 under both walking conditions. However, reliability and validity metrics for certain kinematic parameters, particularly pronation and supination angles, were not specified. Table 1 and Figure 1 present the parameters with units, corresponding definitions from the device and previous study, and graphical illustrations.

### 2.3. Experimental Procedures

In a clinical setting, gait assessment was conducted on a treadmill in accordance with routine clinical practice. For the clinical group (C group), their treating podiatrist selected the appropriate size of the DSPro^®^ insole for each participant. The IMU sensor chip was then fitted into the insole, which was subsequently placed into the participant’s own shoes. Once the preparation was complete, participants stood stationary on a treadmill and waited for the podiatrist to initiate the experiment on the mobile application. Participants waited for 1–2 s and walked on the treadmill for 2–3 min at the self-selected comfortable walking speed to complete the data recording. Participants were instructed not to use the treadmill handrails or any walking aids during data collection.

The control group (H group) was assessed overground in a university corridor, where treadmill facilities were not available for standardized data collection. For each participant, the correct size of the DSPro^®^ insole was selected and placed into participant’s own shoes. The experiments were conducted in a 70 m corridor, where each participant was required to walk back and forth for 2–3 min of data collection. At the end of each trial, in both testing environments, participants were instructed to stand still and remain stationary until the result reports became available.

### 2.4. Data Analysis

Statistical analyses were conducted using the built-in tools of GraphPad Prism10. Spatiotemporal gait parameters obtained from the DSPro^®^ were first categorized based on the availability of bilateral (left and right foot) data. For parameters without bilateral data, both C and H groups were initially subjected to normality testing using the Shapiro–Wilk test. The significance ρ-value was set at the level of 0.05. The Mann–Whitney test was employed to assess between-group differences when the data did not meet the assumption of normality. If the data were normally distributed, an unpaired *t*-test with Welch’s correction was used to determine statistical significance.

For spatiotemporal parameters with bilateral measurements, a two-factor linear mixed-effects model estimated by restricted maximum likelihood (REML) was fitted with “Group” (C group vs. H group) as a between-subject fixed factor, “Foot side” (left vs. right) as a within-subject repeated factor, and random intercepts for subjects. The main effects and interaction effects were tested. Significant interactions were followed by Sidak-adjusted simple effects analyses to examine both group effect and foot-side effect. Simple effects analysis involves isolating one factor, either group or foot side, to determine whether the other factor has a significant influence on the results. When the group factor is constant, the analysis examines left and right foot differences within groups to identify foot-side significance. Conversely, when the foot-side factor is fixed, it assesses between-group differences for the same foot, helping distinguish the normal and abnormal gait by identifying significance on the foot side. This approach enabled more precise interpretation of interaction effects.

## 3. Results

### 3.1. Results of Participant Recruitment

The control group comprised 28 individuals, including 20 males and 8 females (mean age: 28 ± 7 years; mean height: 175 ± 9 cm; mean weight: 70 ± 15 kg) without known foot complaints, recruited from a local university. While the clinical group consisted of 39 individuals including 19 males and 20 females (mean age: 59 ± 18 years; mean height: 168 ± 9 cm; mean weight: 79 ± 18 kg). Among the clinical participants, 16 reported foot pain, localized to the forefoot (1st, 4/5th metatarsophalangeal joint, foot digits), midfoot (midfoot osteoarthritis, dorsal midfoot, plantar arch), or ankle (medial, lateral, anterolateral ankle, ankle osteoarthritis, tarsal tunnel, and deltoid ligament). Additionally, 21 participants exhibited a decreased range of motion in the ankle joint, and 12 exhibited a decreased range of motion in the subtalar joint. Some participants presented with multiple conditions above concurrently.

### 3.2. Overview of Parameters and Statistical Approaches

Among the 22 spatiotemporal parameters analyzed, four parameters, including symmetry, double stance ratio, cadence, and speed, were derived as averages from both feet, while the remaining 18 parameters contained data specific to each foot. Table 2 details the mean and standard deviation values for each parameter in both the C group and H group, along with the ρ results of the corresponding significance. The following sections will present the results based on different statistical analyses.

#### 3.2.1. Significant Analysis Using Mann–Whitney, Unpaired *t*-Test

Of the parameters without foot-specific data, only two parameters, double stance ratio (ρ = 0.0428) and speed (ρ = 0.0003), exhibited significant between-group effects. Specifically, the C group demonstrated a longer average double stance ratio (12.83 ± 2.01%) compared to the H group (11.92 ± 1.57%), and a lower average walking speed, C group (3.76 ± 0.93 km/h) and H group (4.55 ± 0.60 km/h).

#### 3.2.2. Significant Analysis Using Mixed Effects Model

Among the parameters with data specific to each foot, significant group effects were observed for six spatiotemporal parameters, including stance ratio (ρ = 0.0459, F(1,65) = 4.141), swing ratio (ρ = 0.0459, F(1,65) = 4.141), loading time (ρ = 0.0008, F(1,65) = 12.41), and stride length (ρ < 0.0001, F(1,65) = 31.15). Additionally, both clearance (foot-side effect: ρ = 0.0025, F(1,65) = 9.904; group effect: ρ = 0.0022, F(1,65) = 10.19), and foot progression angle (foot-side effect: ρ < 0.0001, F(1,65) = 32.92; group effect: ρ = 0.0220, F(1,65) = 5.503) demonstrated significant effects for both group and foot side, but without a significant interaction effects.

In contrast, five spatiotemporal parameters showed interaction effect, including stance time (Interaction effects: ρ = 0.0276, F(1,65) = 5.076), stride time (Interaction effects: ρ = 0.0174, F(1,65) = 5.957), pronation and supination (PS) angle at toe off (Interaction effects: ρ = 0.0118, F(1,65) = 6.707), steppage angle (Interaction effects: ρ = 0.0017, F(1,65) = 10.74) and propulsion angle (Interaction effects: ρ = 0.0040, F(1,65) = 8.924) showed significant interaction effects, suggesting that main effects alone are insufficient to explain the observed differences. Thus, simple effects analysis was necessary to determine whether differences were driven by group or foot.

#### 3.2.3. Simple Effects Analysis

Five spatiotemporal parameters were found to exhibit significant interaction effects. Thus, further investigation via simple effects analysis was necessary. Table 3 summarizes mean differences and corresponding 95% confidence intervals (95% CI) results from the five spatiotemporal parameters with significant interaction effects. Only two parameters, including steppage angle and propulsion angle, showed a significant group effect, whereas the remaining parameters demonstrated significant differences in only one foot.

Both steppage, and propulsion angles showed clear between-group differences for both feet. The steppage angle was significantly reduced in both feet with the difference between groups for the left foot (−9.15°) being greater than that for the right foot (−6.12°). Similar trends were also observed at propulsion angle, with the right foot (−9.94°) showing a greater group difference than the left foot (−5.64°). Notably, these angles also demonstrated foot-side asymmetry particularly within the H group in steppage angle (mean difference = 4.28°, 95% CI [2.66, 5.89], ρ < 0.0001) and in propulsion angle (mean difference = −2.79°, 95% CI [−5.30, −0.28], ρ = 0.0265).

Six gait parameters showed significant group effects in the previous analysis; however, it remains unclear whether these effects were driven by bilateral or unilateral foot. Table 4 summarizes mean differences and corresponding 95% CI results from six spatiotemporal parameters without significant interaction effects. In the between-group comparison, significant group differences were founded on the left foot for stance and swing ratio. Specifically, participants in the C group spent a greater proportion of the gait cycle in stance and a smaller proportion in swing compared to the H group on the left side (left stance ratio: mean difference = 1.25%, 95% CI [0.10, 2.40], ρ = 0.0309; left swing ratio: mean difference = −1.25%, 95% CI [−2.40, −0.10], ρ = 0.0309), whereas no significant differences were found on the right foot in comparison of C and H group (both ρ = 0.4210).

In contrast, both the loading time and stride length showed significant differences between groups for bilateral foot, with bilaterally reduced values in the C group. Notably, for the loading time, the between-group difference was greater on the right foot than on the left (left mean difference = −9.76 ms, 95% CI [−19.46, −0.07], ρ = 0.0481; right mean difference = −17.08, 95% CI [26.78, −7.39], ρ = 0.0002). In addition, the stride length showed minimal left–right differences between groups, with both feet in the C group exhibiting a reduction of approximately 0.25 m.

Clearance and foot progression angle both demonstrated within-group significance and between-group significance. Within-group comparison for clearance showed a significant difference in the H group, with the right foot exhibiting 0.49 cm greater clearance than the left. In contrast, the C group showed only a 0.16 cm average difference between feet. Between-group contrasts showed higher clearance in right foot in the C group (mean difference = 0.69 cm, 95% CI [0.25, 1.14], ρ = 0.0011), with no significance difference in left foot (ρ = 0.1253). Within-group comparison for foot progression angle exhibited significance for both groups (ρ = 0.0002 and 0.0004, respectively). Between-group comparison indicated that the C group showed greater left-foot toe out (mean difference = 4.06°, 95% CI [0.03–8.09], ρ = 0.0477), but without significant difference in the right (ρ = 0.0894).

## 4. Discussion

This study utilized a commercially available IMU-based device, DSPro^®^, in both clinical and non-clinical settings to determine which spatiotemporal gait parameters can significantly differentiate populations presenting for clinical examination from those recruited from the broader community. Based on the results, 10 spatiotemporal parameters were identified as significantly differentiating the two populations. However, it should be noted that the two groups were assessed under different walking conditions, and the recruited participants had a wide age range (the mean age for clinical group was 59 ± 18 years, and mean age of control group was 28 ± 7 years), both of which have been known to produce biomechanical differences in gait parameters. Therefore, the observed between-group differences may reflect a combination of clinical characteristics, walking conditions, and age differences rather than clinical status alone. The biomechanical implications underlying these statistically significant quantitative parameters need further investigation in future studies.

### 4.1. Potential Biomechanical Mechanisms

**Figure 2:**
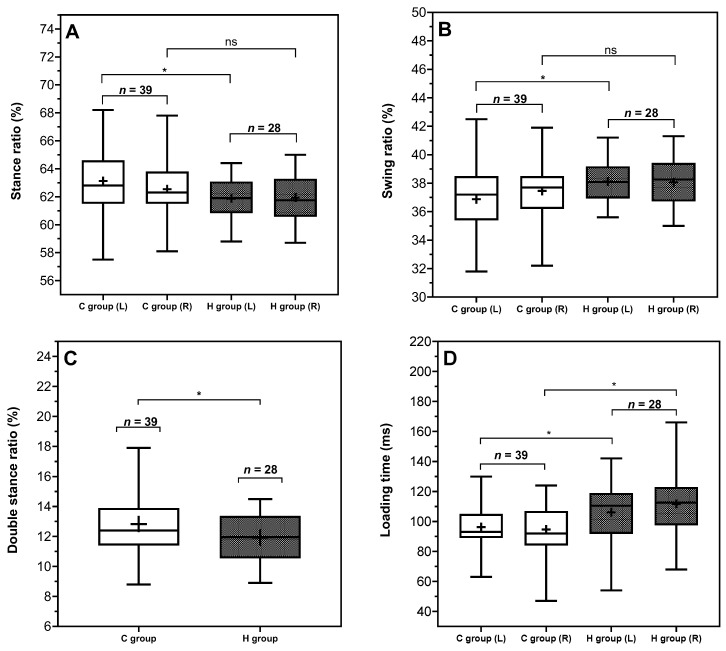
Comparison between C group and H group on gait spatial parameters. (**A**): stance ratio, (**B**): swing ratio, (**C**): double stance ratio, (**D**): loading time. Indicators (*) shown in the figures represent significant group effects identified for the corresponding foot side. The levels of significance are indicated as follows: * (*p* < 0.05).

Significant differences in temporal and symmetry parameters, including the ratios of stance, swing, double stance, and loading time, were observed between the clinical group and the control group. Notably, as illustrated in Figure 2, the clinical group demonstrated increased variability in these parameters, as indicated by the wider standard deviations observed in bilateral foot data compared to the control group. Specifically, the group presenting for clinical assessment appeared to be characterized by conservative temporal pattern. For instance, the stance ratio was greater in the C group (mean value for left foot: 63%, and right foot: 62%) compared to the H group (mean value for left foot: 61%, and right foot: 61%), with a similar trend observed in the double stance ratio (C group: 12.83 ± 2.01%, H group: 11.92 ± 1.57%). Moreover, the relative decrease in loading time by approximately 12% aligned with a reduced swing ratio in the C group may also indicate that these individuals may, intentionally or unintentionally, shorten single-leg support intervals. Considering that foot loading initiates immediately after the end of double stance with the body weight shifting onto the single supporting limb, these observed temporal adjustments may represent altered temporal organization of gait.

**Figure 3:**
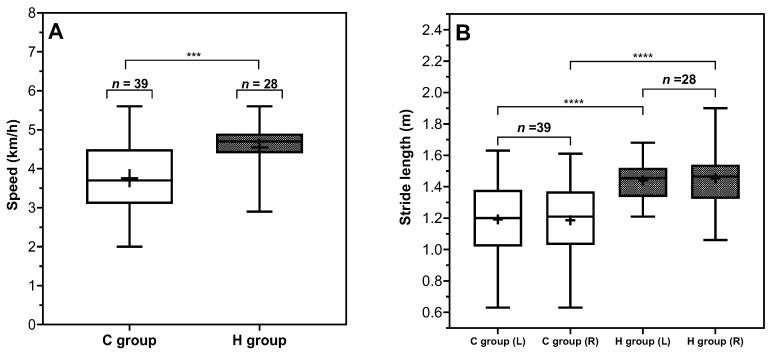
Comparison between C group and H group on gait spatial parameters. *(***A**): Speed, *(***B**): stride length. Indicators (*) shown in the figures represent significant group effects identified for the corresponding foot side. The levels of significance are indicated as follows: *** (*p* < 0.001), and **** (*p* < 0.0001). Insufficient foot dorsiflexion and plantarflexion represent common biomechanical characteristics in abnormal gait. Specifically, in Figure 4, the steppage angle was significantly lower in the C group than in H group. The control population contacted the ground with the foot more dorsiflexed at approximately 25–30° on average while in contrast, the population in the C group exhibited a much flatter heel strike, around 18–20°, indicating reduced 30% dorsiflexion. Meanwhile, within the C group, the propulsion angle was significantly lower, at approximately 47–48°, roughly 8–10° less than that observed in the H group. Reduced foot angle in the sagittal plane may be compatible with altered ankle motion patterns, although this requires further research verification.

Spatial parameters such as gait speed and stride length are influenced by temporal parameters. Consequently, alterations in temporal parameters are typically accompanied by corresponding changes in these spatial parameters. As illustrated in Figure 3, the results indicated a slower walking speed (approximately 0.8 km/h reduction) in the C group (3.76 ± 0.93 km/h) compared to the H group (4.55 ± 0.60 km/h). However, cadence in C group did not differ significantly between groups, maintaining a similar pattern and range of standard deviation to the H group. This phenomenon may imply that the observed reduction in gait speed might be due to a decrease in stride length rather than changes in step frequency. Stride length measurements evidence this interpretation, showing an average reduction of approximately 0.25 m in the C group (1.19 m) relative to the H group (1.44 m), reflecting a 17% reduction in the stride length. From a biomechanics point of view, individuals might have shorter stride length, as has been reported in populations experiencing lower-limb discomfort [23]. Additionally, shorter strides may be associated with altered balance strategies [24]. The consistency observed between stride length and gait speed reductions (17% decrease) across both parameters was consistent with this interpretation.

Foot progression angle and foot clearance are two key spatial parameters that characterize foot motion during the swing phase and may provide insight into compensatory gait adaptations [25]. Clinical presentations exhibited significantly greater foot progression angles, ranging from approximately 8 to 12 degrees compared to 4 to 9 degrees in the H group, despite both groups showing some natural bilateral asymmetry. This increased angle rotation of the foot may reflect an altered foot placement pattern. In addition, foot clearance was also significantly increased in the C group, with values averaging 1.9 to 2.1 cm, approximately 0.5 to 0.7 cm higher than in the control group. From a biomechanical perspective, this combination of increased foot progression angle and increased foot clearance may reflect a modified swing phase strategy often associated with impaired dorsiflexion control [26]. In particular, a larger foot progression angle during swing may be associated with proximal joint adaptations, a compensatory mechanism typically employed to accommodate a plantarflexed ankle or to avoid toe drag [27,28]. This altered kinematic pattern facilitates greater clearance by lifting the foot higher than usual, thereby reducing the risk of tripping and enhancing overall safety during gait. Such parameter combinations can be used to explain biomechanical behavior, suggesting that angle parameters may provide additional descriptive information, and highlighting the clinical value of foot angle measurements in detecting changes in gait patterns.

**Figure 4:**
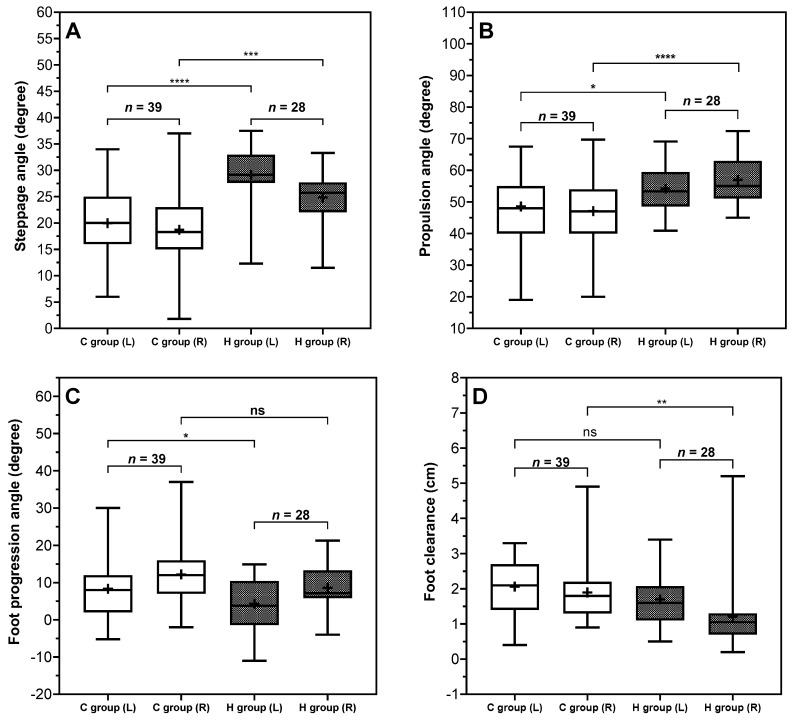
Comparison between C group and H group on gait spatial parameters. *(***A**): Pitch angle at heel strike, *(***B**): pitch angle at toe off, *(***C**): foot progression angle, *(***D**): foot clearance. The positive and negative sign for foot progression angle means abduction (+). Indicators (*) shown in the figures represent significant group effects identified for the corresponding foot side. The levels of significance are indicated as follows: * (*p* < 0.05), ** (*p* < 0.01), *** (*p* < 0.001), and **** (*p* < 0.0001).

### 4.2. Factors Influencing Results

Difference in walking conditions and mean age of participants between groups may have influenced the findings of the study.

Walking conditions (overground vs. treadmill) can affect gait parameters [29]. Previous study suggests that treadmill walking may induce a more cautious behavior compared with overground walking [30], which may alter spatiotemporal parameters. For instance, walking speed during overground walking is often faster than during treadmill walking [30,31,32]. Malatesta et al. [31] found that preferred walking speed during overground walking (1.45 m/s for older cohorts and 1.37 m/s for young cohorts) was higher than during treadmill walking (1.31 m/s for older cohorts and 1.25 m/s for young cohorts). Similarly, study [32] reported that walking speed during overground walking was 0.04 ± 0.06 m/s faster than treadmill walking. Similar findings of gait speed were also reported in the present study, with an approximately mean 0.8 km/h (0.22 m/s) reduction in treadmill walking when compared to overground walking. In addition, the present study observed a 1.25% increase in stance ratio, a 0.91% increase in double stance ratio, a 1.25% decreased in swing ratio, and an average reduction of 0.26 m in stride length during treadmill walking in clinical group. These findings were consistent with previous reports indicating that treadmill walking has been associated with approximately 1% increases in stance and double support durations and a 1% reduced in swing durations [31]. Furthermore, the study [32] reported greater stride length during overground walking compared with treadmill walking when treadmill speed was set to the average overground walking speed. Beyond spatiotemporal parameters, walking conditions have also been shown to influence kinematics parameters, including a reduced heel strike pitch angle during treadmill walking [31] and decreased foot clearance [33] compared with overground walking. The present study, treadmill walking in the clinical group was associated with an approximately reduction of six to nine degrees in steppage angle. However, foot clearance increased by approximately 0.37 to 0.69 cm during treadmill walking in clinical group. This finding warrants further investigation in future research. A recent systematic review [34] further concluded that spatiotemporal outcomes are largely comparable between treadmill and overground walking, but biomechanical differences in kinematics parameters persist and warrant caution.

Age is a well-established determinant of gait biomechanics and represents an important potential confounder in the present study. The clinical group had a substantially higher mean age (59 ± 18, years) than the non-clinical group (28 ± 7, years), which may have contributed to observed differences in spatiotemporal and kinematics parameters. The previous literature consistently demonstrated that increasing age was associated with more conservative temporal strategy [29], including increased stance time and single and double support time, and decreased swing phase, step length, and stride length [35,36]. Many spatiotemporal parameters were speed-dependent, as slower self-selected speed in older cohorts can amplify apparent between-group differences. Beyond spatiotemporal parameters, kinematics parameters also showed significant differences across age cohorts. Older adults demonstrated significantly greater toe out angle [35] and smaller steppage angle [36,37]. A recent systematic review reported reduced ankle plantarflexion and power generation during late stance in older adults [38]. Furthermore, previous studies have reported reduced minimum toe clearance in older adults [37].

Accordingly, the findings should be interpreted with caution, as the observed effects in the present study should be interpreted as reflecting a combination of clinical characteristics, walking-condition effects, and age-related gait adaptations.

### 4.3. Clinical Application and Significance

The findings from this study have provided preliminary evidence regarding which spatiotemporal parameters differ between groups presenting for clinical examination and those from the broader community when using IMU-based system. These results can highlight parameters that may warrant closer attention in clinical gait assessment. However, as this study was cross-sectional in design, the findings did not establish relationships with specific foot pathologies. In addition, the identified parameters should be interpreted as descriptive differentiators between groups, which may inform future longitudinal or interventional investigations. The present results also demonstrate the capacity of wearable IMU-based insoles to generate comprehensive quantitative gait metrics in routine clinical settings. While these tools may offer objective measurement support, further research is required to determine their clinical adaptation in guiding diagnosis and monitoring intervention outcomes.

Beyond the conventional temporal metrics, our results would suggest that foot angle measurements provide additional clinical insights into gait abnormalities. Specifically, the steppage, and propulsion angles and foot progression angles demonstrated significant capability distinguishing gait pattern in different populations, while the PS angle also effectively reflected bilateral foot differences. This finding can hold considerable clinical importance, as traditional gait analysis tools used in clinical settings typically required complex setups involving camera-based video recordings to identify foot angles at key gait events in different anatomical planes. However, such setups rarely captured foot progression angle effectively, given the difficulty in positioning cameras along the transverse plane. The capacity of wearable IMU-based insoles to directly measure these angular parameters thus offers a valuable and practical alternative, facilitating comprehensive gait assessment in routine clinical environments without extensive instrumentation.

## 5. Conclusions

This study employed a commercially available IMU-based wearable insole system (DSPro^®^) to compare spatiotemporal gait parameters between individuals presenting for clinical examination and a non-clinical population. Among the 22 gait parameters analyzed, 10 spatiotemporal parameters, including stance ratio, swing ratio, double stance ratio, gait speed, loading time, stride length, foot progression angle, clearance, steppage, and propulsion angles, demonstrated statistically significant group difference (all ρ ≤ 0.0459). For parameters measured bilaterally, statistical significance was observed in at least one foot.

These findings indicated that selected temporal, spatiotemporal, and kinematics parameters differed between the two groups assessed in this study. However, given the cross-sectional design, the use of different walking environments, and the wide age range of participants, including the mean age for clinical group was 59 ± 18 years, and mean age of control group was 28 ± 7 years, the results should be interpreted with caution. The observed differences may reflect a combination of clinical characteristics, age-relate effects, and walking environment influences. Therefore, the present findings are descriptive in nature and do not provide treatment guidance. The ability of these parameters to support clinical decision-making, predict outcomes, or evaluate the effect of interventions requires further investigation. However, the results can contribute to the growing body of evidence supporting the feasibility of IMU-based wearable insoles in clinical gait assessment.

## 6. Limitations

This study acknowledges limitations that should be considered when interpreting the findings. First, the population recruited from the clinical setting had a higher mean age (59 ± 18, years) compared with the control group (28 ± 7, years). Age can affect gait, particularly spatiotemporal parameters [29,39]. Future studies should consider age-matched groups to minimize this potential effect. Secondly, the testing environments of the treadmill for the clinical group and overground for the control group may have influenced the results. Thirdly, all participants wore their personal footwear during data collection. Although the existing literature [40] has highlighted the impact of different footwear types on running gait kinematics, the influence of varying shoe types on walking gait spatiotemporal parameters remains unclear. An additional limitation concerns the kinematic validation of the DSPro^®^ system, as the pronation and supination angles have not been validated against a gold standard system. Therefore, caution is warranted when interpreting these metrics. Furthermore, future research should investigate the biomechanical implications of spatiotemporal parameters associated with lower limb or foot presentations in clinical settings. Such efforts will facilitate the integration of IMU-based wearable insoles as a more effective tool for examination in clinical practice.

## Figures and Tables

**Table 1:** The parameters measured by DSPro^®.^

Walking Profile	Speed (km/h) Cadence (step/min) Symmetry (percentage %) Double stance (percentage %)	Stride length (m) Stride duration (ms) Stance time/ratio (ms, percentage %) Swing time/ratio (ms, percentage %)
Foot Kinematics	Gait line (ms) Pronation and supination angles (degree)	Foot progression angle (degree) Circumduction (cm)
Advanced Parameters	Steppage and propulsion angle (degree)	Clearance (cm)

Speed: antero-posterior speed of the estimated center of mass; Cadence: step frequency per minute; Symmetry: percentage calculated on the symmetry of stance time on both feet; Double stance: sum of double support time; Stride length: duration of the contact phase and oscillation phases; Stride duration: time between initial heel strike and final heel strike of the same side; Stance time: time between initial heel strike and toe off of the same side; Swing time: time between toe off and final heel strike of the same side; Gait line: trajectory of the center of pressure during the three stance phases, including loading time (time between initial heel strike and flat foot in of the same side), flatfoot time (time between flat foot in and flat foot out of the same side) and propulsion time (time between flat foot out and toe off of the same side); Pronation and supination angles: provides the angle of pronation/supination during the four points of the contact phase: heel strike, foot flat, heel off and toe off; Foot progression angle: angle of opening of the patient’s step; Circumduction: measures the lateral distance made by the foot during the flight phase; Steppage and propulsion angle: angle formed between the foot and the ground at heel strike/toe off; Clearance: minimum height between the toes and the ground during the oscillating phase. The parameter definitions list above are derived from the DSPro device and its published Supplementary Materials in the study [19].

**Table 2:** Statistical results for spatiotemporal parameters using Mann–Whitney, unpaired *t*-test, and mixed-effects model.

Spatiotemporal Parameters with Unit	C Group (Mean, SD)	H Group (Mean, SD)	Mann–Whitney Test/Unpaired *t*-Test (*ρ*-Value < 0.05)	Mixed-Effects Analysis (*ρ*-Value < 0.05)
Symmetry (%)	94.18 ± 6.34	95.75 ± 2.90	0.6225	
Double stance ratio (%)	12.83 ± 2.01	11.92 ± 1.57	0.0428 *	
Cadence (steps per second)	104.3 ± 11.07	105.0 ± 10.16	0.5250	
Speed (km/h)	3.76 ± 0.93	4.55 ± 0.60	0.0003 *	
Stance ratio (left/right) (%)	63.13 ± 2.37/62.54 ± 2.14	61.88 ± 1.53/61.94 ± 1.76		Foot-side effect: ρ = 0.2570, F (1,65) = 1.308, Group effect: ρ = 0.0459 *, F (1,65) = 4.141, Interaction effects: ρ = 0.1637, F (1,65) = 1.984
Swing ratio (left/right) (%)	36.87 ± 2.37/37.46 ± 2.14	38.12 ± 1.53/38.06 ± 1.76		Foot-side effect: ρ = 0.2570, F (1,65) = 1.308, Group effect: ρ = 0.0459 *, F (1,65) = 4.141, Interaction effects: ρ = 0.1637, F (1,65) = 1.984
Stance time (left/right) (ms)	728.0 ± 112.52/716.6 ± 107.61	691.7 ± 50.04/694.2 ± 52.44		Foot-side effect: ρ = 0.1564, F (1,65) = 2.056, Group effect: ρ = 0.1966, F (1,65) = 1.702, Interaction effects: ρ = 0.0276 *, F (1,65) = 5.076
Swing time (left/right) (ms)	422.2 ± 39.61/426.1 ± 41.19	425.2 ± 21.88/426.0 ± 25.80		Foot-side effect: ρ = 0.37, F (1,65) = 0.8148, Group effect: ρ = 0.8584, F (1,65) = 0.03210, Interaction effects: ρ = 0.5635, F (1,65) = 0.3372
Stride time (left/right) (ms)	1149 ± 143.79/1142 ± 141.15	1117 ± 63.69/1120 ± 66.86		Foot-side effect: ρ = 0.4044, F (1,65) = 0.7043, Group effect: ρ = 0.3549, F (1,65) = 0.8682, Interaction effects: ρ = 0.0174 *, F (1,65) = 5.957
Propulsion time (left/right) (ms)	227.8 ± 48.90/218.8 ± 40.45	218.7 ± 67.51/220.5 ± 69.60		Foot-side effect: ρ = 0.4637, F (1,65) = 0.5434, Group effect: ρ = 0.7801, F (1,65) = 0.07858, Interaction effects: ρ = 0.2751, F (1,65) = 1.212
Foot flat time (left/right) (ms)	400.4 ± 101.31/402.5 ± 95.73	367.8 ± 89.74/364.7 ± 90.20		Foot-side effect: ρ = 0.9240, F (1,65) = 0.009173, Group effect: ρ = 0.1353, F (1,65) = 2.287, Interaction effects: ρ = 0.6322, F (1,65) = 0.2313
Loading time (left/right) (ms)	96.31 ± 13.57/94.67 ± 15.19	106.1 ± 20.30/111.8 ± 20.00		Foot-side effect: ρ = 0.3073, F (1,65) = 1.059, Group effect: ρ = 0.0008 *, F (1,65) = 12.41, Interaction effects: ρ = 0.0666, F (1,65) = 3.480
Stride length (left/right) (m)	1.19 ± 0.22/1.19 ± 0.21	1.44 ± 0.11/1.45 ± 0.16		Foot-side effect: ρ = 0.6005, F (1,65) = 0.2770, Group effect: ρ < 0.0001 *, F (1,65) = 31.15, Interaction effects: ρ = 0.2491, F (1,65) = 1.353
Circumduction (left/right) (cm)	2.66 ± 1.28/2.49 ± 1.48	2 ± 1.59/2.18 ± 1.80		Foot-side effect: ρ = 0.9310, F (1,65) = 0.007547, Group effect: ρ = 0.1847, F (1,65) = 1.797, Interaction effects: ρ = 0.1644, F (1,65) = 1.977
Clearance (left/right) (cm)	2.06 ± 0.72/1.90 ± 0.76	1.70 ± 0.70/1.20 ± 0.95		Foot-side effect: ρ = 0.0025 *, F (1,65) = 9.904, Group effect: ρ = 0.0022 *, F (1,65) = 10.19, Interaction effects: ρ = 0.1202, F (1,65) = 2.480
Pronation and supination angle at heel strike (left/right) (degree)	−14.56 ± 5.84/−15.41 ± 5.41	−13.21 ± 6.06/−14.68 ± 6.04		Foot-side effect: ρ = 0.0408 *, F (1,65) = 4.357, Group effect: ρ = 0.4442, F (1,65) = 0.5926, Interaction effects: ρ = 0.5785, F (1,65) = 0.3118
Pronation and supination angle at foot flat (left/right) (degree)	−8.69 ± 4.17/−8.87 ± 3.85	−8.14 ± 3.15/−7 ± 3.61		Foot-side effect: ρ = 0.1776, F (1,65) = 1.858, Group effect: ρ = 0.1727, F (1,65) = 1.901, Interaction effects: ρ = 0.0658, F (1,65) = 3.501
Pronation and supination angle at heel off (left/right) (degree)	−7.61 ± 3.77/−7.33 ± 3.42	−7.25 ± 2.92/−5.71 ± 2.97		Foot-side effect: ρ = 0.0096 *, F (1,65) = 7.131, Group effect: ρ = 0.2013, F (1,65) = 1.667, Interaction effects: ρ = 0.0701, F (1,65) = 3.392
Pronation and supination angle at toe off (left/right) (degree)	−4.26 ± 4.46/−2.67 ± 4.49	−4.5 ± 4.29/−0.23 ± 4.21		Foot-side effect: ρ < 0.0001 *, F (1,65) = 32.04, Group effect: ρ = 0.2632, F (1,65) = 1.274, Interaction effects: ρ = 0.0118 *, F (1,65) = 6.707
Foot progression angle (left/right) (degree)	8.35 ± 7.92/12.2 ± 7.51	4.29 ± 6.42/8.61 ± 5.68		Foot-side effect: ρ < 0.0001 *, F (1,65) = 32.92, Group effect: ρ = 0.0220 *, F (1,65) = 5.503, Interaction effects: ρ = 0.7421, F (1,65) = 0.1093
Steppage angle (left/right) (degree)	19.97 ± 6.58/18.72 ± 7.35	29.12 ± 5.03/24.85 ± 5.08		Foot-side effect: ρ < 0.0001 *, F (1,65) = 35.69, Group effect: ρ < 0.0001 *, F (1,65) = 25.89, Interaction effects: ρ = 0.0017 *, F (1,65) = 10.74
Propulsion angle (left/right) (degree)	48.56 ± 10.25/47.06 ± 10.93	54.2 ± 7.10/56.99 ± 7.23		Foot-side effect: ρ = 0.3741, F (1,65) = 0.8011, Group effect: ρ = 0.0009 *, F (1,65) = 12.20, Interaction effects: ρ = 0.0040 *, F (1,65) = 8.924

Note: Spatiotemporal parameters with statistically significant effects are highlighted with a * sign.

**Table 3:** Simple effects analysis for parameters which has significance on interaction effects.

Spatiotemporal Parameters	ρ Value (Group)	Mean Difference (Group)	95% CI of Difference (Group)	ρ Value (Foot Side)	Mean Difference (Foot Side)	95% CI of Difference (Foot Side)
Stance time (ms)	0.0116 */0.8345	11.41/−2.54	2.25 to 20.57/−13.35 to 8.275	0.2117/0.5461	36.32/22.38	−15.06 to 87.7/−29 to 73.8
Stride time (ms)	0.0270 */0.5070	6.95/−3.40	0.68 to 13.22/−10.79 to 4.00	0.4684/0.7006	32.42/22.08	−33.93 to 98.78/−44.27 to 88.44
Pronation and supination angle at toe off (degree)	0.0406 */<0.0001 *	−1.59/−4.27	−3.12 to −0.06/−6.08 to −2.46	0.9695/0.0564	0.244/−2.44	−2.25 to 2.74/−4.93 to 0.05
Steppage angle (degree)	0.0802/<0.0001 *	1.25/4.28	−0.12 to 2.61/2.66 to 5.89	<0.0001 */0.0003 *	−9.15/−6.12	−12.71 to −5.60/−9.68 to −2.57
Propulsion angle (degree)	0.2087/0.0265 *	1.51/−2.79	−0.62 to 3.63/−5.30 to −0.28	0.0347 */<0.0001 *	−5.64/−9.94	−10.94 to −0.34/−15.24 to −4.64

Note: Indicators (*) marked under “group” represent the significance of differences between feet within the same group. Left: complaints group; right: control group. Indicators marked under “foot side” represent the significance of differences for the same foot side between groups. Left: left foot; right: right foot. The sign indicates the result of the subtraction between feet (left-right) or groups (C-H). A sign appearing under the Group category denotes whether the value for the left foot is higher (+) or lower (−) than the right foot within the same group. A sign appearing under the Foot Side category indicates whether the value in the complaints (C) group is higher (+) or lower (−) than the control (H) group for the same foot.

**Table 4:** Mean difference and 95% confidence interval results for parameters which had no significance on interaction effects.

Spatiotemporal Parameters	ρ Value (Group)	Mean Difference (Group)	95% CI of Difference (Group)	ρ Value (Foot Side)	Mean Difference (Foot Side)	95% CI of Difference (Foot Side)
Stance ratio (%)	0.1025/0.9811	0.58/−0.06	−0.09 to 1.26/−0.86 to 0.74	0.0309 */0.4210	1.25/0.60	0.10 to 2.40/−0.55 to 1.75
Swing ratio (%)	0.1034/0.9812	−0.58/0.06	−1.26 to 0.09/−0.74 to 0.86	0.0309 */0.4210	−1.25/−0.60	−2.40 to −0.10/−1.75 to 0.55
Loading time (ms)	0.7695/0.1207	1.64/−5.68	−4.17 to 7.45/−12.53 to 1.17	0.0481 */0.0002 *	−9.76/−17.08	−19.46 to −0.07/−26.78 to −7.39
Stride length (m)	0.8587/0.4705	0.0053/−0.01	−0.02 to 0.03/−0.04 to 0.02	<0.0001 */< 0.0001 *	−0.25/−0.27	−0.35 to −0.14/−0.37 to −0.16
Clearance (cm)	0.4046/0.0058 *	0.16/0.49	−0.14 to 0.47/0.13 to 0.86	0.1253/0.0011 *	0.37/0.69	−0.08 to 0.81/0.25 to 1.14
Foot progression angle (degree)	0.0002 */0.0004 *	−3.85/−4.33	−5.96 to −1.74/−6.82 to −1.84	0.0477 */0.0894	4.06/3.59	0.03 to 8.09/−0.44 to 7.62

Note: Indicators (*) marked under “group” represent the significance of differences between feet within the same group. Left: complaints group; right: control group. Indicators marked under “foot side” represent the significance of differences for the same foot side between groups. Left: left foot; right: right foot. The sign indicates the result of the subtraction between feet (left-right) or groups (C-H). A sign appearing under the Group category denotes whether the value for the left foot is higher (+) or lower (−) than the right foot within the same group. A sign appearing under the Foot Side category indicates whether the value in the complaints (C) group is higher (+) or lower (−) than the control (H) group for the same foot.

## Data Availability

The data is unavailable due to ethical restrictions.

## Supplementary Materials

The following supporting information can be downloaded at: https://www.mdpi.com/article/10.3390/s26061802/s1, Supplementary file S1.
